# High expression of small nucleolar host gene RNA may predict poor prognosis of Hepatocellular carcinoma, based on systematic reviews and meta-analyses

**DOI:** 10.1186/s12885-024-12590-2

**Published:** 2024-09-05

**Authors:** Sheng-qi Du, Ya-Tong Liu, Fen Yang, Pei-xue Wang, Jun Zhang

**Affiliations:** 1 Department of Gastroenterology, The First People’s Hospital of Jingzhou, The First Affiliated Hospital of Yangtze University, Jingzhou, 434000 Hubei China; 2 Department of Emergency, The First People’s Hospital of Jingzhou, The First Affiliated Hospital of Yangtze University, Jingzhou, 434000 Hubei China

**Keywords:** lncRNA, SNHG, Hepatocellular carcinoma, Prognosis, Meta-analysis

## Abstract

**Background:**

The prognosis of patients with hepatocellular cancer is substantially correlated with the abnormal expression of growing long non-coding RNA small nucleolar host gene RNA (SNHG) families in liver cancer tissues. This study aimed to examine the relationship between SNHG expression and liver cancer prognosis.

**Methods:**

After searching six internet databases, pertinent manuscripts were found based on inclusion and exclusion criteria. To determine whether SNHG expression levels affect liver cancer prognosis, raw data were collected and hazard ratios (HRs) and odds ratios (ORs) were calculated. The results were examined for potential publication bias using the sensitivity analysis and Beeg’s test.

**Results:**

Most SNHG family members were up-regulated in liver cancer tissues. High SNHG expression predicts poor liver cancer outcomes of, including overall survival (OS) (HR: 1.697, 95% confidence interval [CI]: 1.373–2.021), especially SNHG5 (the HR of OS is 4.74, 95%CI range from 1.35 to 6.64), progression-free survival (HR: 1.85, 95% CI: 1.25–2.73), tumor, node, metastasis (TNM) stage (OR: 1.696, 95% CI: 1.436–2.005), lymph node metastasis (OR: 2.383, 95% CI: 1.098–5.173), and tumor size (OR: 1363, 95% CI: 1.165–1.595). The OS results were found to be reliable and robust, as indicated by the sensitivity analysis. Additionally, Beeg’s test demonstrated the absence of any potential publication bias for each result.

**Conclusion:**

In liver cancer tissues, most SNHGs are highly expressed, which may signal poor prognosis. SNHG has the potential to be an intriguing predictive marker and a prospective therapeutic target for liver cancer.

## Introduction

Cancer is the primary cause of human mortality, resulting in significant mental and physical suffering for individuals and an extensive financial strain on the global community on an annual basis [1]. Globally, approximately 10 million cancer-related fatalities and 19.8 million newly diagnosed cancer patients were reported in 2020, as per the 2021 global cancer statistics [2]. Among all newly diagnosed cancers, liver cancer ranks fifth among new cases among men and ninth among women [3, 4]. The 5-year survival rate remains unsatisfactory, although the application of targeted therapy, immunotherapy, radiotherapy, and chemotherapy has enhanced the survival benefits of patients with liver cancer to a certain extent [5, 6]. Several investigators are endeavoring to identify novel therapeutic targets and prognostic indicators [7, 8].

As molecular biology and high-throughput sequencing have advanced, long non-coding RNAs (lncRNAs), a family of small molecule nucleotides without protein-coding activities, are now recognized as a leading risk factor for liver cancer [9, 10]. LncRNAs influence coding RNA expression at the transcriptional, post-transcriptional translation, and post-translational modification levels and directly or indirectly interfere with the cell cycle, proliferation, immigration, invasion, and apoptosis of tumor cells by acting on downstream genes or signal cascades through sponging with microRNAs [11–13]. The prognosis of liver cancer was substantially correlated with the growth of aberrantly expressed long noncoding genes [14, 15]. Zhao et al. [16], for instance, demonstrated that small nucleolar host gene RNA (SNHG) 7 (SNHG7) may enhance the proliferation, immigration, and metastasis of hepatocellular carcinoma (HCC) cells by increasing forkhead box K2 expression through sponging and decreasing miR-122-5p. According to Kou et al. [17], NEAT1 regulates Bax, Bcl-2, and epidermal growth factor receptors to help HCC cells proliferate, invade, and suppress apoptosis.

The lncRNA family SNHG includes dozens of family members [18–20]. Additional studies show that SNHG regulates liver cancer cell proliferation, migration, metastasis, and apoptosis and is linked to liver cancer prognosis [21, 22]. Many researchers have examined the link between SNHG expression and liver cancer prognosis because SNHG may act as a promising target for treatment and prognostic marker [23, 24]. This study endeavors to conduct a meta-analysis to comprehensively investigate the association between the expression level of SNHG and the prognosis of liver cancer, in light of the limited sample size of a single study and the conflicting findings and conclusions of various studies.

## Materials and methods

### Literature search strategy

We thoroughly searched six electronic databases—China National Knowledge Infrastructure (CNKI), Web of Science, Google Scholar, Cochrane Library, Embase, and PubMed—to find pertinent literature. The following are the detailed search terms: “liver neoplasm” OR “liver cancer” OR “hepatocellular carcinoma” OR “hepatic carcinoma” OR “liver cell carcinoma” OR “liver tumor” OR “hepatoma” OR “hepatocarcinoma” OR “malignant hepatoma” OR “liver malignancy” OR “HCC” AND “small nucleolar RNA host lncRNA” OR “long non-coding RNA SNHG” OR “non-coding RNA SNHG” OR “small nucleolar RNA host gene” OR “snoRNA host gene” OR “lncRNA SNHG” OR “SNHG.” In addition to these search terms, we also checked reference lists of relevant studies to find other potentially relevant literature. The publication year of the literature is restricted to March 1, 2023, from database inception.

### Inclusion and exclusion criteria

The original literature must satisfy the subsequent inclusion criteria for incorporation into this investigation: (1) The SNHG level was detected using clear detection methods. (2) The cancer patients were categorized into two groups: the SNHG low expression group and the SNHG high expression group, as determined using the SNHG level. (3) The primary goal of the initial investigation was to explore the relationship between SNHG expression and liver prognosis. (4) The thesis’s research quality must satisfy the established standards. (5) Provide sufficient and available data. Original documents meeting the following criteria will be excluded: (1) The research object was not a population. (2) The data were unavailable or insufficient. (3) Non-English literature. (4) Reviews, meta-analyses, case reports, and meeting abstracts.

### Quality assessment of included literature

Two researchers independently assessed the quality of the included studies using the Newcastle-Ottawa Quality Assessment Scale (NOS). NOS is an evaluation tool widely used in non-randomized controlled studies [25]. This mainly includes the following three major projects: (1) Selection of research objects. (2) Comparability between groups. (3) Exposure/outcome evaluation. There are a total of 8 sub-items with a total of 9 points based on star ratings, and we will assign each study a quality score based on its performance in these areas. If two researchers are different regarding the scoring of the same original document, they may either engage in a discussion or request that a third researcher negotiate a resolution. A score between 6 and 9 was suggestive of excellent and appropriate for this study’s inclusion. A score between 0 and 5 was regarded as low quality and discarded.

### Data extraction

This meta-analysis comprised original literature from which two researchers independently extracted the first author’s name, publication year, number of cases, SNHG level cut-off value, and detection method. The hazard ratio (HR) and 95% confidence interval (CI) were also retrieved to assess the relationship between SNHG expression and liver cancer prognosis. When survival curves are the sole information provided in the original literature, HR values with 95% CI were derived indirectly using the Engauge 4.0 version software [26]. To investigate the association between SNHG expression and HCC clinicopathological parameters, including distant metastasis (DM), lymph node metastasis (LNM), tumor, node, metastasis (TNM) stage, and tumor size, the number of occurrences and total number of events of each clinicopathological parameter were also extracted.

### Statistical analysis

Stata SE 12.0 and Revman 5.4.0 were used in this investigation. The relationship between SNHG expression and the survival prognosis in HCC patients was evaluated using the pooled HR with 95% CI results. The correlation between the clinicopathological features (LNM, DM, and TNM stage) of patients with HCC and SNHG expression and, was investigated using the combined OR with 95% CI results. The significance of heterogeneity was evaluated using I-square (*I*^*2*^) and p-value (p). When I^2^ ≤ 50% and *p* ≥ 0.05, we do not anticipate significant heterogeneity in the results and would apply the fixed-effect model; when I^2^ > 50% and *p* < 0.05, we consider the results to be significantly heterogeneous and would apply subgroup analysis and the random-effect model. Sensitivity analysis was conducted to investigate the robustness and dependability of the findings. To ascertain whether publication bias or other types of bias affected the original study’s findings, the Beeg’s test was used.

## Result

### The included publications’ fundamental characteristics

**Fig. 1:**
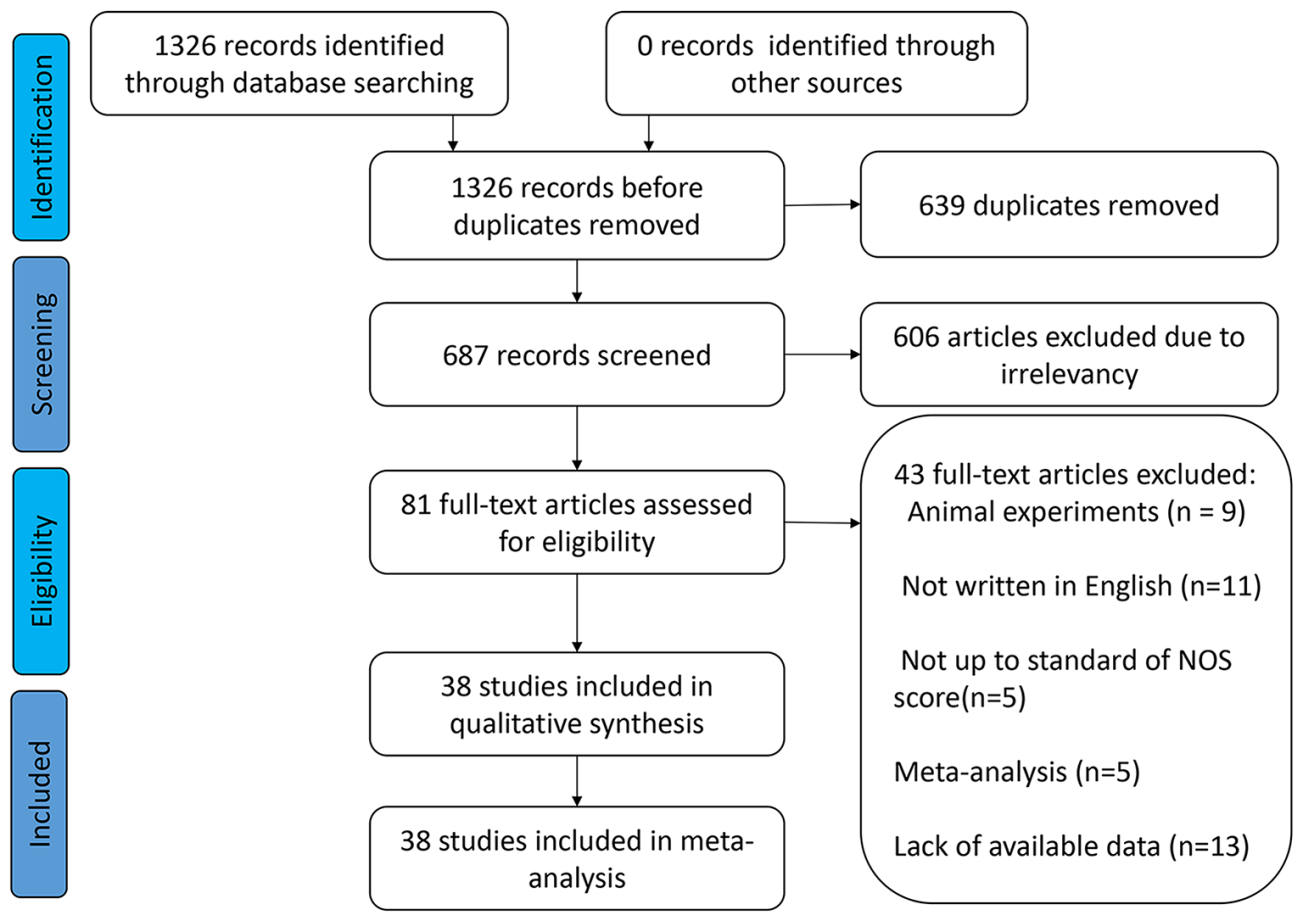
The process for including appropriate articles

A comprehensive search of six internet databases yielded 1326 original articles. In total, 639 duplicate publications and 609 original studies were eliminated because they did not examine the relationship between SNHG expression and liver cancer prognosis. Among the remaining 78 publications, 13 studies had insufficient data, 9 studies did not study the population, 11 literatures were not written in English, 5 articles were meta-analyzed, and the research quality of 5 studies was not up to standard. This study included 38 pieces of original evidence from 2917 patients [16, 23, 27–62]. These 38 publications included only Chinese patients. Except for decreased SNHG2 (also named as GAS5) expression in liver cancer tissues [30, 31, 63], most of the SNHG family members were highly expressed in liver cancer tissues (Fig. 1 and Table 1). Most studies examined the relationship between SNHG expression and liver cancer prognosis, with 30 to 160 patients and NOS scores of 6 to 9 (Table 2).

**Table 1:** Basic features of the publications included in this meta-analysis (*n* = 38)

Author and year	lncSNHG	sample size	expression level	detected method	cut-off value	refence gene	prognostic index	HR with 95%CI	HR extraction	follow-up-month	NOS score
Meng FZ [21]	SNHG1	115	upregulation	qRT-PCR	median	β-actin	OS	1.999 (1.302–3.06)	paper	60	9
Zhang M [28]	SNHG1	82	upregulation	qRT-PCR	median	GAPDH	OS	2.14 (1.12–4.11)	survival curve	60	8^a^
							DFS	2.22 (1.25–3.94)			
Hu LT 2015 [30]	GAS5 (SNHG2)	30	downregulation	qRT-PCR	mean	GAPDH	OS	0.48 (0.169–1.37)	survival curve	22	7^a^
Chang L [29]	GAS5 (SNHG2)	60	downregulation	qRT-PCR	mean	GAPDH	OS	0.307 (0.113–0.415)	paper	60	9
Tu ZQ [31]	GAS5 (SNHG2)	71	downregulation	qRT-PCR	mean	GAPDH	OS	0.417 (0.244–0.617)	paper	60	9
Zhang T 2015 [39]	SNHG3	144	upregulation	qRT-PCR	not reported	β-actin	OS	3.464 (1.820–6.594)	paper	60	8^d^
							RFS	2.134 (1.311–3.474)	paper	60	8^d^
							DFS	1.97 (1.19–3.28)	survival curve	60	8^d^
Zhang PF 2018 [38]	SNHG3	70	upregulation	qRT-PCR	mean	GAPDH	OS	1.94 (1.17–3.22)	survival curve	24	7^a^
Li YR 2018 [34]	SNHG5	48	upregulation	qRT-PCR	median	β-actin	OS	4.74 (1.350–6.640)	paper	36	9
							RFS	3.690 (1.229–11.082)	paper	36	9
Cao C 2016 [32]	SNHG6	160	upregulation	qRT-PCR	mean	β-actin	OS	1.832 (1.032–3.253)	paper	60	9
Fan XX [23]	SNHG6	40	upregulation	qRT-PCR	mean	GAPDH	OS	1.71 (0.49–5.96)	survival curve	100	8^a^
							PFS	1.78 (0.69–4.62)	survival curve	100	8^a^
Xie YT [36]	SNHG7	80	upregulation	qRT-PCR	mean	GAPDH	OS	1.89 (1.16–3.08)	survival curve	60	6^be^
Shen A [35]	SNHG7	100	upregulation	qRT-PCR	median	GAPDH	OS	2.584 (1.621–3.880)	paper	60	9
							PFS	1.86 (1.21–2.86)	paper	60	9
Yang X [37]	SNHG7	80	upregulation	qRT-PCR	median	GAPDH	OS	2.87 (1.53–5.39)	survival curve	60	7^ab^
Zhao ZB 2021 [16]	SNHG7	30	upregulation	qRT-PCR	mean	GAPDH	OS	3.25 (0.8-13.21)	survival curve	60	8^a^
Feng SG [33]	SNHG9	40	upregulation	qRT-PCR	mean	GAPDH	OS	2.44 (0.44–13.39)	survival curve	60	8^a^
Lan T [45]	SNHG10	64	upregulation	qRT-PCR	mean	GAPDH	OS	1.144 (1.042–1.256)	paper	60	9
Huang W [42]	SNHG11	57	upregulation	qRT-PCR	mean	GAPDH	OS	1.68 (0.85–3.30)	survival curve	60	7^ab^
Lan T [44]	SNHG12	48	upregulation	qRT-PCR	median	GAPDH	OS	2.28 (0.88–5.88)	survival curve	48	7^ab^
							RFS	2.24 (1.01-5)	survival curve	48	7^ab^
Wang X [50]	SNHG13	62	upregulation	qRT-PCR	mean	GAPDH	OS	3.63 (2.01–6.58)	survival curve	44	7^ab^
Liu Y [48]	SNHG13	66	upregulation	qRT-PCR	median	GAPDH	OS	2.3 (1.02–5.18)	survival curve	60	7^ab^
Ma X [49]	SNHG13	52	upregulation	qRT-PCR	mean	β-actin	not reported	NA	NA	NA	7^ab^
Yuan SX [54]	DANCR (SNHG13)	135	upregulation	qRT-PCR	median	GAPDH	OS	2.757 (1.379–5.514)	paper	48	9
							RFS	2.228 (1.359–3.653)	paper	48	9
Zhang H [56]	SNHG14	40	upregulation	qRT-PCR	mean	GAPDH	OS	1.77 (0.61–5.1)	survival curve	36	8^a^
Xu XY [52]	SNHG14	55	upregulation	qRT-PCR	mean	GAPDH	not reported	NA	NA	NA	7^c^
Liao ZB [46]	SNHG14	66	upregulation	qRT-PCR	mean	GAPDH	OS	1.3 (0.67–2.5)	survival curve	120	8
Zhang JH [57]	SNHG15	152	upregulation	qRT-PCR	median	GAPDH	OS	2.247 (1.331–6.255)	paper	66	9
Chen W 2020 [40]	SNHG15	58	upregulation	qRT-PCR	mean	GAPDH	OS	1.64 (0.56–4.83)	survival curve	60	8^a^
Dai W [41]	SNHG15	101	upregulation	qRT-PCR	mean	GAPDH	not reported	NA	NA	NA	7^c^
Jing Z [43]	SNHG16	40	upregulation	qRT-PCR	mean	GAPDH	OS	1.48 (0.71–3.1)	survival curve	96	7
Lin Q 2018 [47]	SNHG16	88	upregulation	qRT-PCR	mean	GAPDH	OS	2.34 (1.04–5.28)	survival curve	60	8^a^
Zhong JH 2019 [58]	SNHG16	108	upregulation	qRT-PCR	median	GAPDH	OS	1.94 (1.07–3.52)	survival curve	60	8^a^
							DFS	1.69 (1.07–2.66)	survival curve	60	
Ye JF [53]	SNHG16	103	upregulation	qRT-PCR	mean	GAPDH	not reported	NA	NA	NA	7^c^
Xie XH [51]	SNHG16	40	upregulation	qRT-PCR	mean	β-actin	not reported	NA	NA	NA	7^c^
Zhu XM [59]	SNHG17	58	upregulation	qRT-PCR	mean	GAPDH	OS	1.426 (0.796–3.434)	paper	60	9
Zhang DY 2016 [55]	SNHG20	144	upregulation	qRT-PCR	median	GAPDH	OS	3.985 (1.981–8.017)		60	9
Liu JX [60]	SNHG20	96	upregulation	qRT-PCR	median	GAPDH	OS	2.79 (1.74–4.48)	survival curve	60	8^a^
Zhang YX [62]	SNHG22	60	upregulation	qRT-PCR	mean	GAPDH	not reported	NA	NA	NA	7^c^
Luo J [61]	MEG8 (SNHG23)	74	upregulation	qRT-PCR	mean	GAPDH	OS	2.29 (1.22–4.32)	survival curve	60	8^a^
							DFS	1.89 (1.11–3.2)	survival curve	60	8^a^

*Note *

^a^not multivariate analysis

^b^Survival curves only, lack of clinicopathological parameters

^c^No follow-up data, only clinicopathological parameters provided

^d^Not report the cutoff-value

^e^Possible other bias due to relatively low sample size

SNHG: small nucleotide host RNA; OS: overall survival; HR: hazard ratio; CI: confidence interval; PFS: progression-free survival; DFS: disease-free survival; RFS: Recurrence free survival. NA: not available. GAPDH: glyceraldehyde-3-phosphate dehydrogenase; qRT-PCR: Quantitative real time polymerase chain reaction; NOS: Newcastle-Ottawa Quality Assessment Scale; survival curve: The original literature only provides survival curves without HR values and 95% CI, and HR values with 95% CI were derived indirectly using the Engauge 4.0 version software; paper: The original literature directly provides HR values and 95% CI

**Table 2:** Quality assessment of eligible studies Newcastle-Ottawa scale (NOS) score

Author and Year	Country	Selection				Comparability	Outcome			Total
		Adequate of case definition	Representativeness of the cases	Selection of Controls	Definition of Controls	Comparability of cases and controls	Ascertainment of exposure	Same method of ascertainment	Non-Response rate	
Meng FZ 2021 [21]	China	*	*	*	*	**	*	*	*	9
Zhang M 2016 [28]	China	*	*	*	*	*	*	*	*	8
Hu LT 2015 [30]	China	*	*	*	*	*	*	*	-	7
Chang L 2016 [29]	China	*	*	*	*	**	*	*	*	9
Tu ZQ 2014 [31]	China	*	*	*	*	**	*	*	*	9
Zhang T 2015 [39]	China	*	*	*	*	*	*	*	*	8
Zhang PF 2018 [38]	China	*	*	*	*	*	*	*	-	7
Li YR 2018 [34]	China	*	*	*	*	**	*	*	*	9
Cao C 2016 [32]	China	*	*	*	*	**	*	*	*	9
Fan XX 2021 [23]	China	*	*	*	*	*	*	*	*	8
Xie YT 2020 [36]	China	-	*	*	*	*	*	*	-	6
Shen A 2020 [35]	China	*	*	*	*	**	*	*	*	9
Yang X 2019 [37]	China	*	*	*	*	*	*	*	-	7
Zhao ZB 2021 [16]	China	*	*	*	*	*	*	*	*	8
Feng SG 2021 [33]	China	*	*	*	*	*	*	*	*	8
Lan T 2019 [45]	China	*	*	*	*	**	*	*	*	9
Huang W 2020 [42]	China	*	*	*	*	*	*	*	-	7
Lan T 2017 [44]	China	*	*	*	*	*	*	*	-	7
Wang X 2020 [50]	China	*	*	*	*	*	*	*	-	7
Liu Y 2020 [48]	China	*	*	*	*	*	*	*	-	7
Ma X 2016 [49]	China	*	*	*	*	*	*	*	-	7
Yuan SX 2016 [54]	China	*	*	*	*	**	*	*	*	9
Zhang H 2020 [56]	China	*	*	*	*	*	*	*	*	8
Xu XY 2020 [52]	China	*	*	*	*	*	*	*	-	7
Liao ZB 2021 [46]	China	*	*	*	*	*	*	*	*	8
Zhang JH 2016 [57]	China	*	*	*	*	**	*	*	*	9
Chen W 2020 [40]	China	*	*	*	*	*	*	*	*	8
Dai W 2019 [41]	China	*	*	*	*	*	*	*	-	7
Jing Z 2020 [43]	China	*	*	*	*	*	*	*	-	7
Lin Q 2018 [47]	China	*	*	*	*	*	*	*	*	8
Zhong JH 2019 [58]	China	*	*	*	*	*	*	*	*	8
Ye JF 2019 [53]	China	*	*	*	*	*	*	*	-	7
Xie XH 2019 [51]	China	*	*	*	*	*	*	*	-	7
Zhu XM 2021 [59]	China	*	*	*	*	**	*	*	*	9
Zhang DY 2016 [55]	China	*	*	*	*	**	*	*	*	9
Liu JX 2017 [60]	China	*	*	*	*	*	*	*	*	8
Zhang YX 2021 [62]	China	*	*	*	*	*	*	*	-	7
Luo J 2021 [61]	China	*	*	*	*	*	*	*	*	8

### Association between the survival prognosis of HCC and SNHG expression

**Fig. 2:**
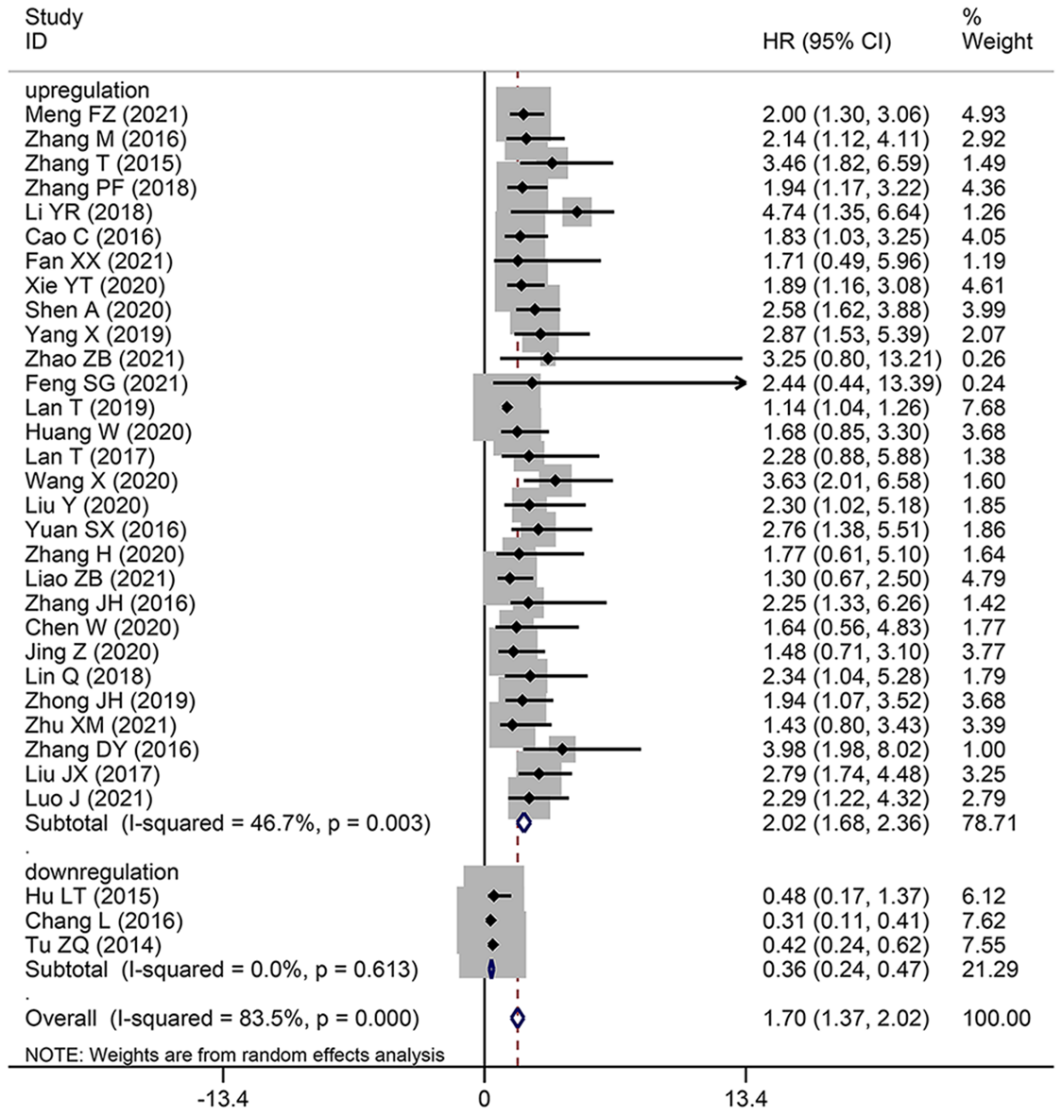
Forest plot showed the correlation between SNHG expression and overall survival (OS) of Hepatocellular carcinoma (HCC). *Note* HR: hazard ratio CI: confidence interval

**Fig. 3:**
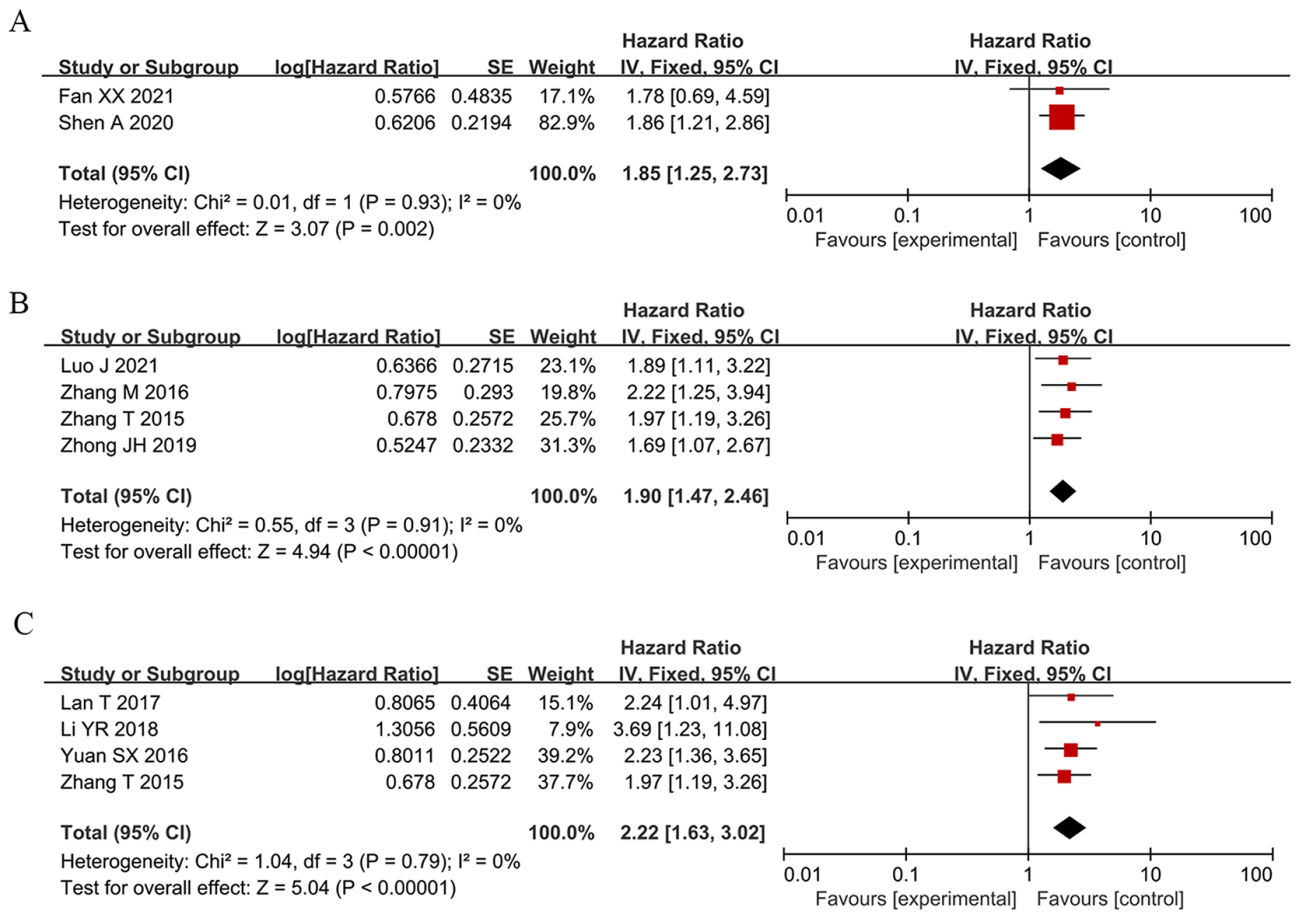
Forest plot showed the correlation between SNHG expression and progress-free survival (PFS), disease-free survival (DFS) and Recurrence free survival (RFS) of Hepatocellular carcinoma (HCC). *Note* (**A**) PFS; (**B**) DFS; (**C**) RFS. HR: hazard ratio CI: confidence interval

To assess the correlation between HCC prognosis and SNHG expression, 32 studies were included in this meta-analysis, covering 2506 patients with HCC. The combination of HR and 95% CI demonstrates a positive and statistically significant relationship between increased SNHG expression and poor prognosis for HCC (HR: 1.697, 95% CI: 1.373–2.021). Subgroup analysis was used in this study because of the significant heterogeneity of the results (*I*^*2*^ = 83.5%, *p* < 0.0001), the inconsistent SNHG expression (increased and decreased level), the mean and median cut-off values, the multivariate and univariate analysis methods, the sample size (< 100 and not < 100), the follow-up month (< 60 and not < 60), and the study quality (NOS score) across different original studies. Pooling HR with 95% CI demonstrated similar favorable correlation between increasing SNHG expression and poor OS in the subgroup of patients with elevated SNHG expression (HR: 1.259, 95% CI: 1.159–1.359), < 9 of NOS score (HR: 1.613, 95% CI: 1.315–1.911), univariate analysis of analysis method (HR: 1.613, 95% CI: 1.315–1.911), median of cut-off value (HR: 2.412, 95% CI: 1.966–2.858), < 100 of sample size (HR: 1.484, 95% CI: 1.131–1.837), not < 100 of sample size (HR: 2.229, 95% CI: 1.744–2.715) (Fig. 2 and Table 3). Furthermore, combining HR and 95% CI reveals a significant positive link between high SNHG expression and poor progression-free survival (PFS) (HR: 1.85, 95% CI: 1.25–2.73) (Fig. 3A), disease-free survival (HR: 1.90, 95% CI: 1.47–2.46) (Fig. 3B), and relapse-free survival (HR: 2.22, 95% CI: 1.63–3.02) (Fig. 3C).

**Table 3:** Pooled HRs of overall survival of hepatocellular carcinoma patients with increased SNHG expression

Subgroup analysis	sample size	No. of studies	Pooled HR (95% CI)		*P*	Heterogeneity	
			Fixed	Random		I^2^(%)	*p* ^− value^
**OS**	32	2506	0.870 (0.795–0.946)	1.697 (1.373–2.021)	< 0.0001	83.5	< 0.0001
**SNHG expression**							
Upregulation	29	2345	1.259 (1.159–1.359)	2.021 (1.677–2.364)	< 0.0001	46.7	0.003
Downregulation	3	161	0.355 (0.240–0.470)	0.355 (0.240–0.470)	< 0.0001	0	0.613
**NOS score**							
9	11	1107	0.819 (0.741–0.897)	1.428 (0.959–2.760)	< 0.0001	92.6	< 0.0001
less than 9	21	1399	1.613 (1.315–1.911)	1.834 (1.446–2.221)	< 0.0001	27.7	0.118
**Analytical method**							
Multivariate analysis	11	1107	0.819 (0.741–0.897)	1.428 (0.959–1.898)	< 0.0001	92.6	< 0.0001
Univariate analysis	21	1399	1.613 (1.315–1.911)	1.834 (1.446–2.221)	< 0.0001	27.7	0.118
**Cut-off value**							
Mean	19	1188	0.822 (0.745–0.899)	1.263 (0.906–1.620)	< 0.0001	86.1	< 0.0001
Median	12	1174	2.412 (1.966–2.858)	2.412 (1.966–2.858)	< 0.0001	0	0.852
Not reported	1	144	3.464 (1.077–5.851)	3.464 (1.077–5.851)	0.004	NA	NA
**Sample size**							
not less than 100	8	1058	2.229 (1.744–2.715)	2.229 (1.744–2.715)	< 0.0001	0	0.788
less than 100	24	1448	0.837 (0.760–0.913)	1.484 (1.131–1.837)	< 0.0001	85	< 0.0001
**Follow-up month**							
not less than 60	25	2073	0.859 (0.783–0.936)	1.643 (1.291–1.994)	< 0.0001	85.3	< 0.0001
less than 60	7	433	1.271 (0.807–1.734)	2.241 (1.094–3.389)	< 0.0001	72.5	0.001
**Refence gene**							
GAPDH	28	2039	0.852 (0.776–0.928)	1.581 (1.245–1.916)	< 0.0001	83.7	< 0.0001
β-actin	4	467	2.211 (1.569–2.853)	2.443 (1.463–3.422)	< 0.0001	42.8	0.155

*Note* OS: overall survival; Random: Random effects; Fixed: Fixed effects; directly: HR was extracted directly from the primary articles; indirectly: HR was extracted indirectly from the primary articles; CI: confidence interval

### The relationship between the TNM stage of HCC and SNHG expression

**Fig. 4:**
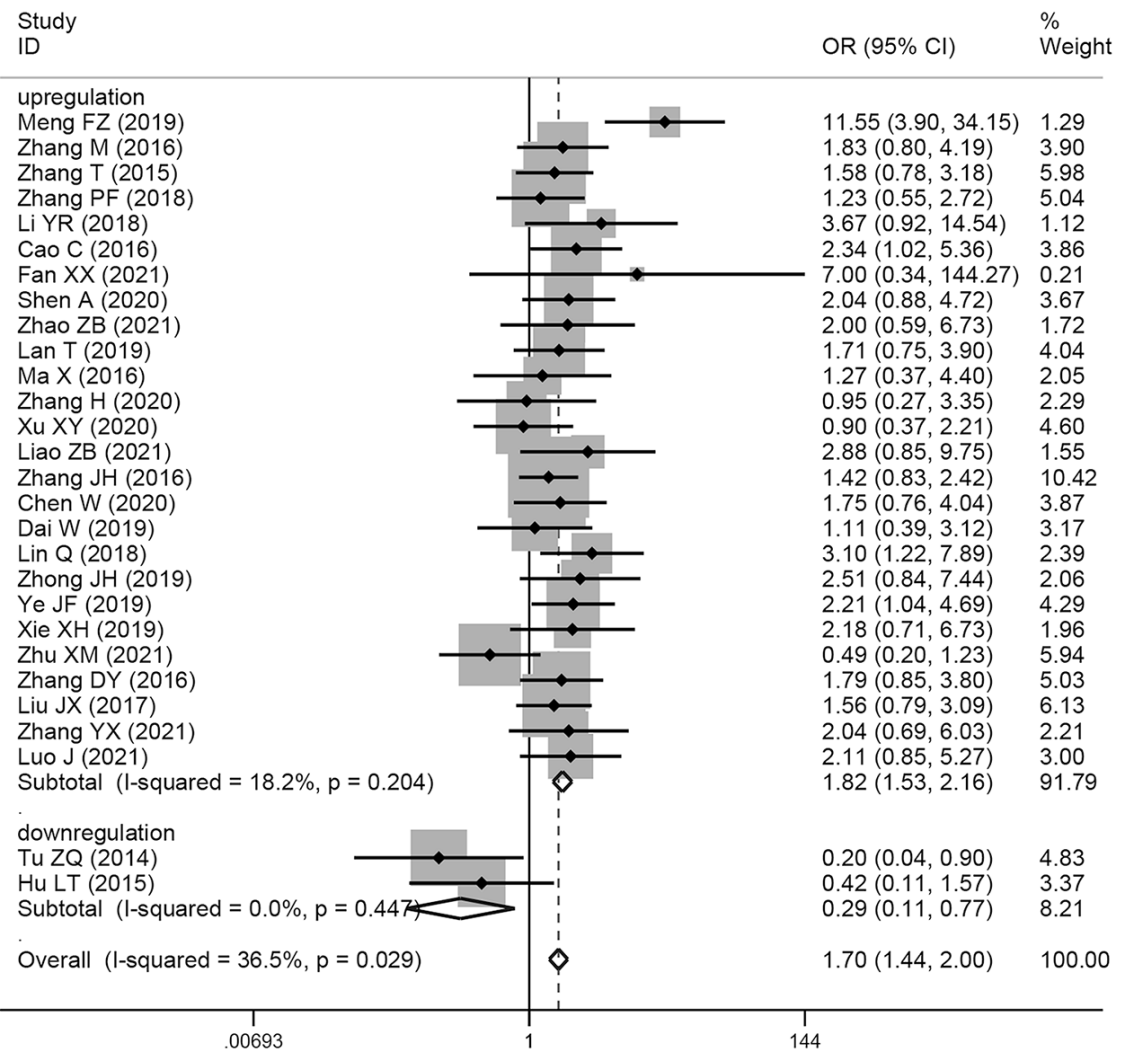
Forest plot showed the correlation between SNHG expression and TNM stage of Hepatocellular carcinoma (HCC). *Note* OR: odds ratio CI: confidence interval

This study examined 28 publications with 2251 patients to determine whether TNM stage and SNHG expression are related. According to the pooled OR with 95% CI values (OR: 1.696, 95% CI: 1.436–2.005) (Fig. 4), an advanced TNM stage has been linked to high SNHG expression. We conducted a subgroup analysis although the overall results’ heterogeneity was negligible (*I*^*2*^ = 36.5%, *p* = 0.029), but heterogeneity is unavoidably caused by different main studies’ varying cut-off values, research quality, and analytical techniques. Subgroup analysis findings showed that elevated SNHG expression (SNHG1, SNHG3, SNHG8, SNHG15, and others) manifested advanced TNM stage (HR: 1.822, 95%CI: 1.534–2.164), while low SNHG expression (SNHG2) manifested advanced stage of TNM (HR: 0.29, 95%CI: 0.109–0.771) (Table 4).

**Table 4:** Pool effects of clinicopathologic characteristics in hepatocellular carcinoma patients with abnormal SNHG expression

Subgroup analysis	sample size	No. of studies	Pooled OR (95% CI)		*P*	Heterogeneity	
			Fixed	Random		I2(%)	*p*-value
**TNM stage**	28	2251	1.696 (1.436–2.005)	1.672 (1.335–2.094)	< 0.0001	36.5	0.029
**NOS_score**							
9	9	912	1.743 (1.341–2.266)	1.698 (0.964–2.991)	0.067	72.6	< 0.0001
less than 9	19	1339	1.665 (1.341–2.067)	1.657 (1.330–2.064)	< 0.0001	0	0.77
**SNHG expression**							
increased	26	2148	1.822 (1.534–2.164)	1.777 (1.457–2.167)	< 0.0001	18.2	0.204
decreased	2	103	0.290 (0.109–0.771)	0.306 (0.113–0.827)	0.02	0	0.447
**LNM**	6	492	2.514 (1.747–3.620)	2.383 (1.098–5.173)	< 0.0001	72.7	0.003
**NOS_score**							
9	3	286	2.293 (0.392–13.421)	2.293 (0.392–13.421)	0.357	88.7	< 0.0001
less than 9	3	206	2.334 (1.351–4.030)	2.314 (1.336–4.008)	0.002	0	0.787
**SNHG expression**							
increased	5	421	3.373 (2.238–5.085)	3.239 (1.831–5.730)	< 0.0001	43.1	0.135
decreased	1	71	0.364 (0.113–1.172)	0.364 (0.113–1.172)	0.09	NA	NA
**DM**	5	518	1.265 (0.846–1.892)	1.415 (0.658–3.042)	0.252	39.4	0.158
**NOS_score**							
9	2	304	1.784 (0.539–5.904)	1.770 (0.070-45.067)	0.343	74.6	0.047
less than 9	3	214	1.203 (0.783–1.849)	1.417 (0.668–3.005)	0.399	35.3	0.213
**Tumor size**	25	2018	1.363 (1.165–1.595)	1.358 (1.093–1.687)	0.006	39.7	0.022
**NOS_score**							
9	9	912	1.339 (1.060–1.690)	1.403 (0.918–2.143)	0.117	63	0.006
less than 9	16	1106	1.384 (1.119–1.711)	1.353 (1.061–1.725)	0.015	17.3	0.255
**SNHG expression**							
increased	23	1915	1.434 (1.221–1.685)	1.433 (1.168–1.758)	< 0.0001	31	0.079
decreased	2	103	0.394 (0.167–0.933)	0.394 (0.167–0.933)	0.034	0	0.95
**Histological grade**	17	1557	1.399 (1.140–1.717)	1.394 (1.135–1.713)	0.001	0	0.843
**NOS_score**							
9	7	741	1.387 (1.030–1.868)	1.383 (1.026–1.864)	0.031	0	0.703
less than 9	10	816	1.410 (1.064–1.870)	1.404 (1.056–1.866)	0.017	0	0.677
**Cut-off value**							
mean	10	776	1.359 (1.019–1.810)	1.351 (1.011–1.805)	0.037	0	0.507
median	6	637	1.482 (1.087–2.020)	1.479 (1.084–2.017)	0.013	0	0.874
not reported	1	144	1.165 (0.489–2.777)	1.165 (0.489–2.777)	0.731	NA	NA
**Depth of invasion**	11	1077	1.911 (1.476–2.475)	1.869 (1.439–2.427)	< 0.0001	0	0.9
**NOS_score**							
9	4	434	1.965 (1.305–2.958)	1.910 (1.263–2.888)	0.001	0	0.601
less than 9	7	643	1.876 (1.344–2.619)	1.842 (1.314–2.581)	< 0.0001	0	0.81
**Cut-off value**							
mean	6	495	2.077 (1.400-3.082)	2.030 (1.364–3.021)	< 0.0001	0	0.899
median	4	438	1.659 (1.159–2.376)	1.651 (1.150–2.369)	0.006	0	0.682
not reported	1	144	3.689 (1.042–13.054)	3.689 (1.042–13.054)	0.043	NA	NA
**Age**	26	2703	1.047 (0.894–1.226)	1.046 (0.893–1.226)	0.567	0	1
**Gender**	27	2136	1 (0.832–1.203)	0.999 (0.829–1.205)	0.994	0	0.97

*Note* TNM: Tumor Node Metastasis, LNM: lymph node metastasis, DM: distant metastasis, CI: confidence interval, No.: number, NA: not applicable

### Association between HCC tumor size and SNHG expression

**Fig. 5:**
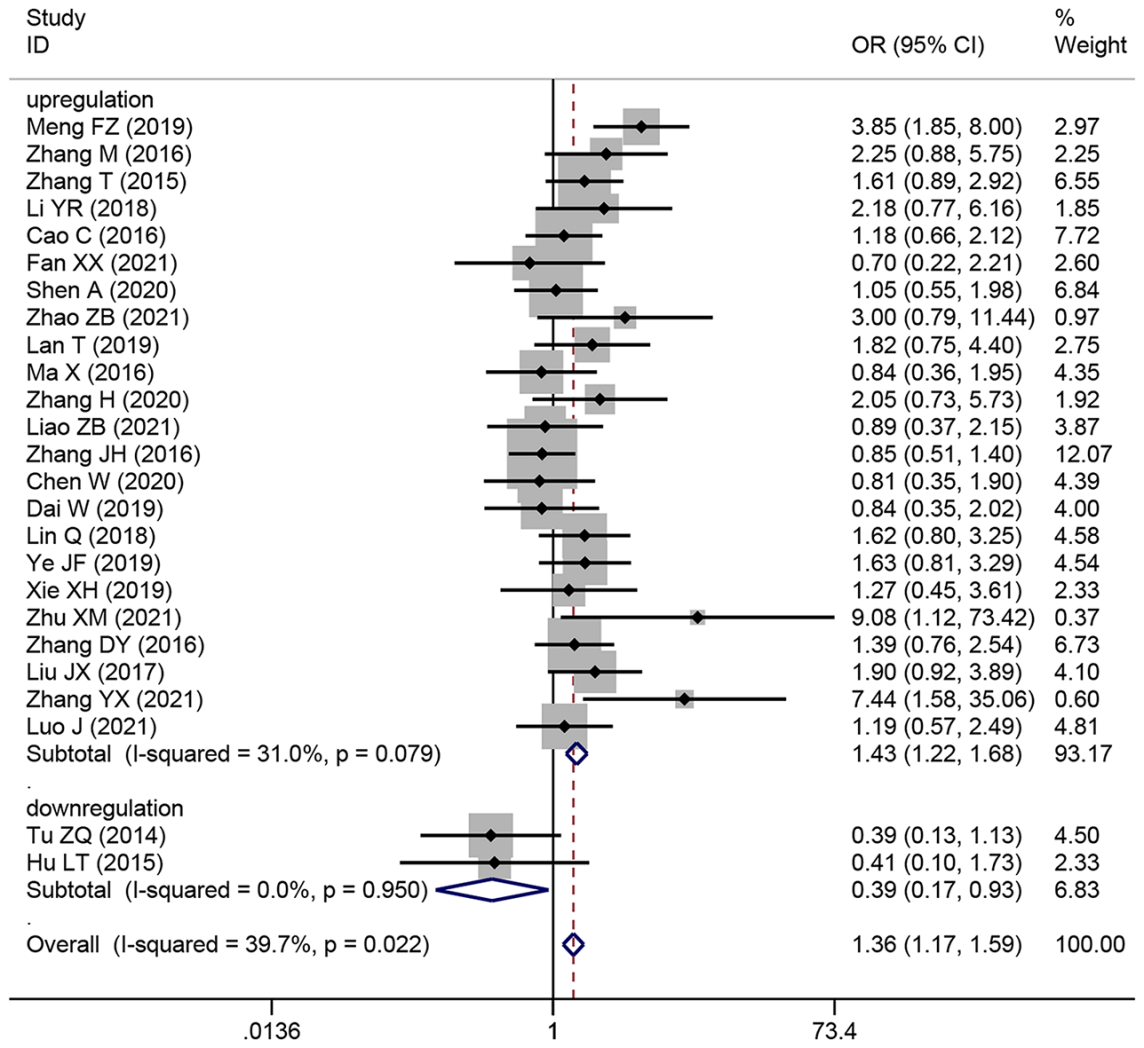
Forest plot showed the correlation between SNHG expression and tumor size of Hepatocellular carcinoma (HCC). *Note* OR: odds ratio CI: confidence interval

This research involved 25 publications that focused on 2018 patients with HCC and examined the relationship between SNHG expression and HCC tumor size. Pooling OR with 95% CI showed a strong substantial relationship between elevated SNHG expression and larger HCC tumors (OR: 1.363, 95% CI: 1.165–1.595) (Fig. 5). The results of subgroup analysis demonstrated that in the subgroup of elevated SNHGs expression (for example SNHG1, SNHG3, SNHG9, SNHG12, SNHG16 et al.), increasing SNHG expression manifesting bigger tumor size (HR: 1.434, 95%CI: 1.221–1.685), in the subgroup of low SNHGs expression (for example SNHG2), increasing SNHG expression manifesting smaller tumor size (HR: 0.394, 95%CI: 0.167–0.933) (Table 4).

### Association between various clinicopathological markers and SNHG expression

**Fig. 6:**
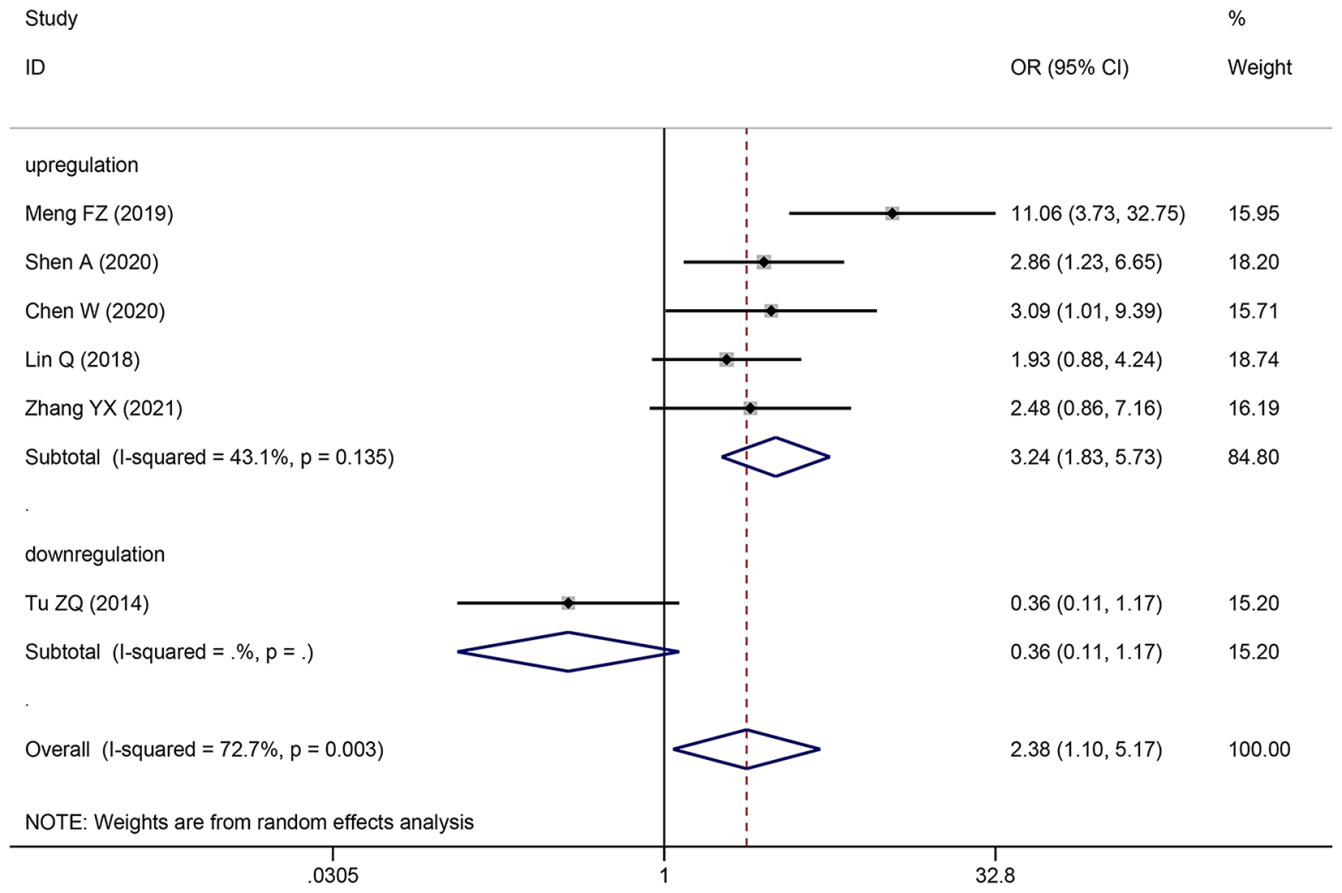
Forest plot showed the correlation between SNHG expression and LNM of Hepatocellular carcinoma (HCC). *Note* OR: odds ratio CI: confidence interval

**Fig. 7:**
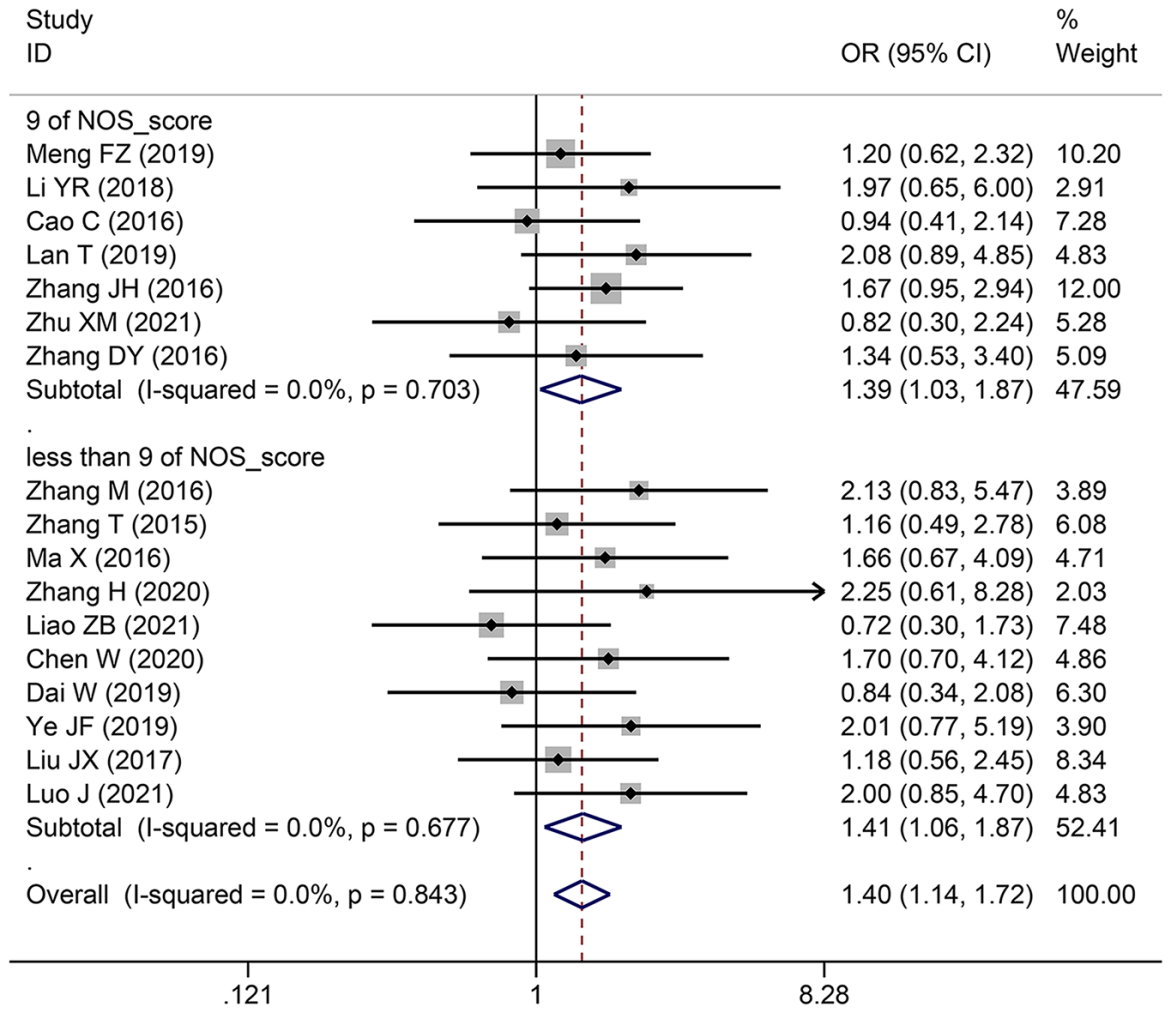
Forest plot showed the correlation between SNHG expression and histological grade of Hepatocellular carcinoma (HCC). *Note* OR: odds ratio CI: confidence interval

**Fig. 8:**
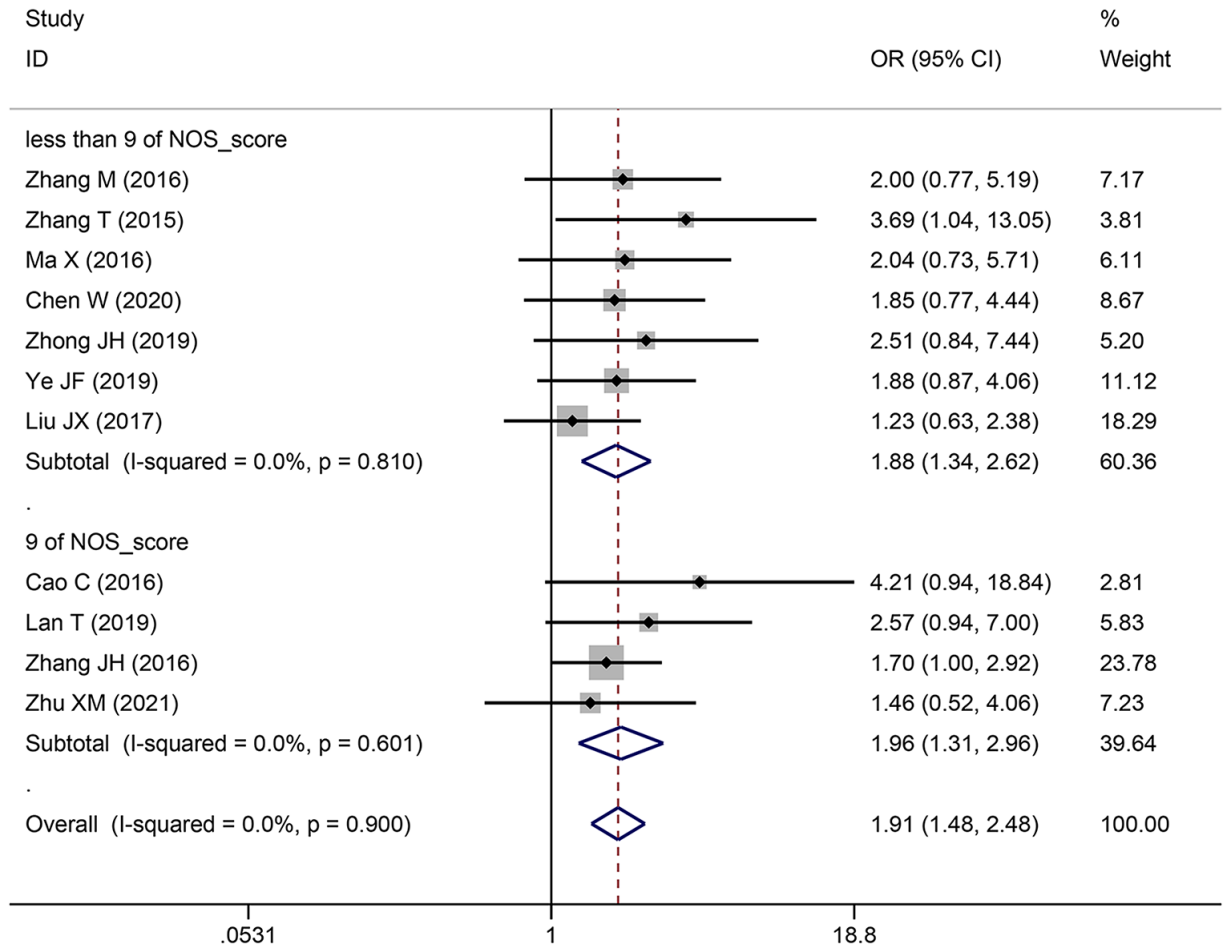
Forest plot showed the correlation between SNHG expression and depth of invasion of Hepatocellular carcinoma (HCC). *Note* OR: odds ratio CI: confidence interval

**Fig. 9:**
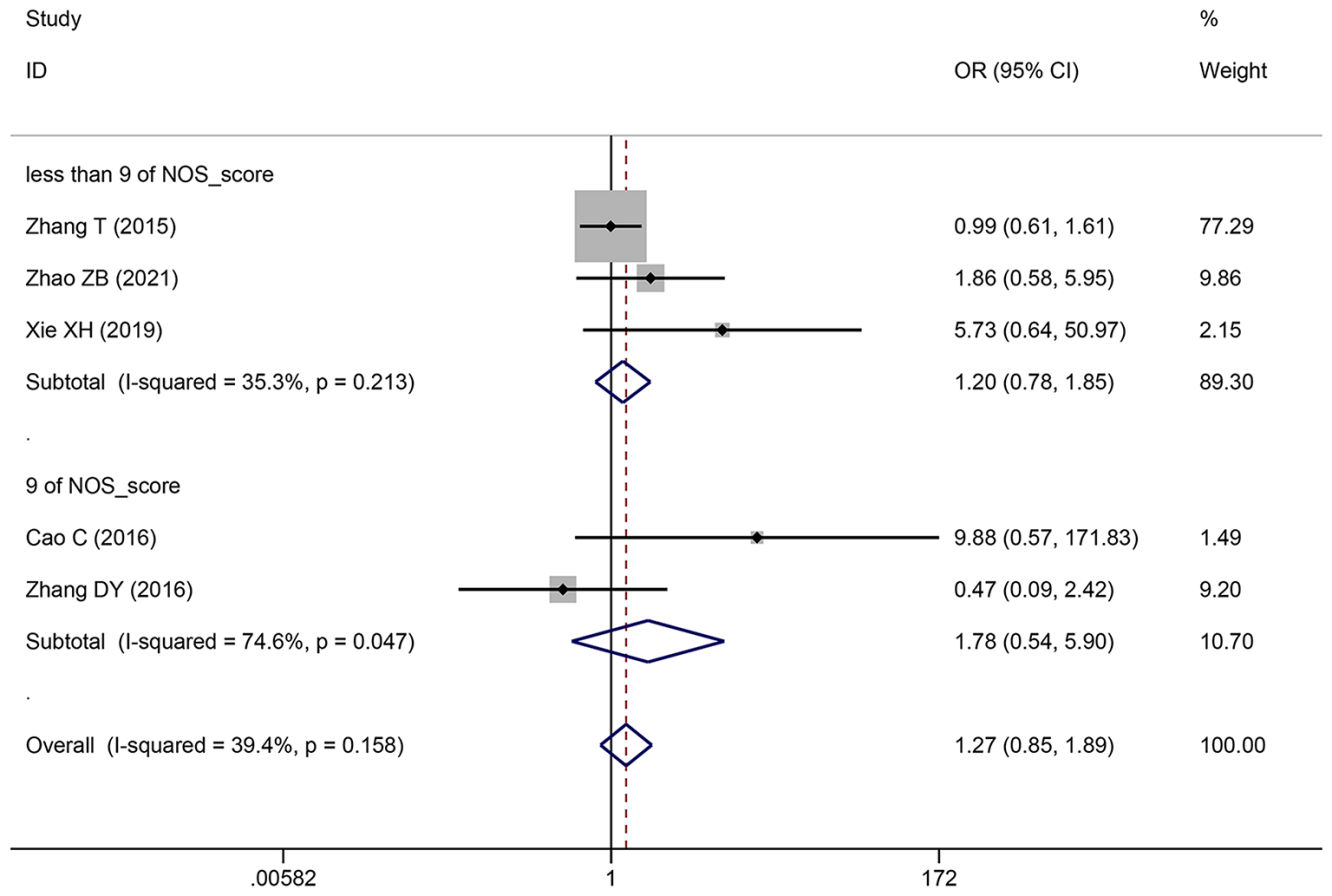
Forest plot showed the correlation between SNHG expression and DM of Hepatocellular carcinoma (HCC). *Note* OR: odds ratio CI: confidence interval

Pooling OR with 95% CI show a significant positive relationship between elevated SNHG expression and easier LNM (OR: 2.383, 95% CI: 1.098–5.173) (Fig. 6), poor histologic status (OR: 1.399, 95% CI: 1.140–1.717) (Fig. 7) and deeper HCC cell invasion (OR: 1.911, 95% CI: 1.476–2.475) (Fig. 8). Simultaneously, DM (OR: 1.265, 95% CI: 0.846–1.892) (Fig. 9), gender (OR: 1, 95% CI: 0.832–1.203), and age (OR: 1.047, 95% CI: 0.894–1.226) were found to have an insignificant relationship with SNHG expression (Table 4).

### Publication bias and sensitivity analysis

**Fig. 10:**
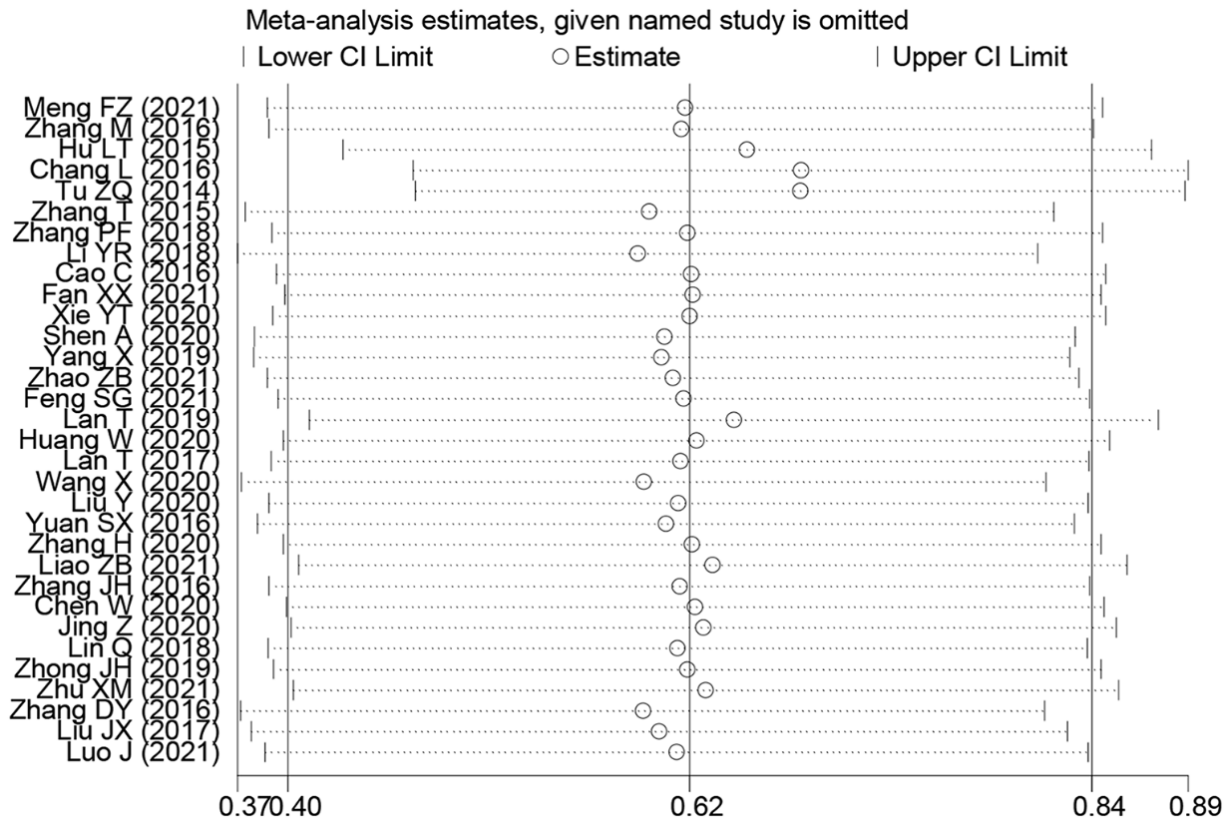
Sensitivity analysis for SNHG expression with overall survival (OS) of Hepatocellular carcinoma (HCC). *Note* HR: hazard ratio, CI: confidence interval

**Fig. 11:**
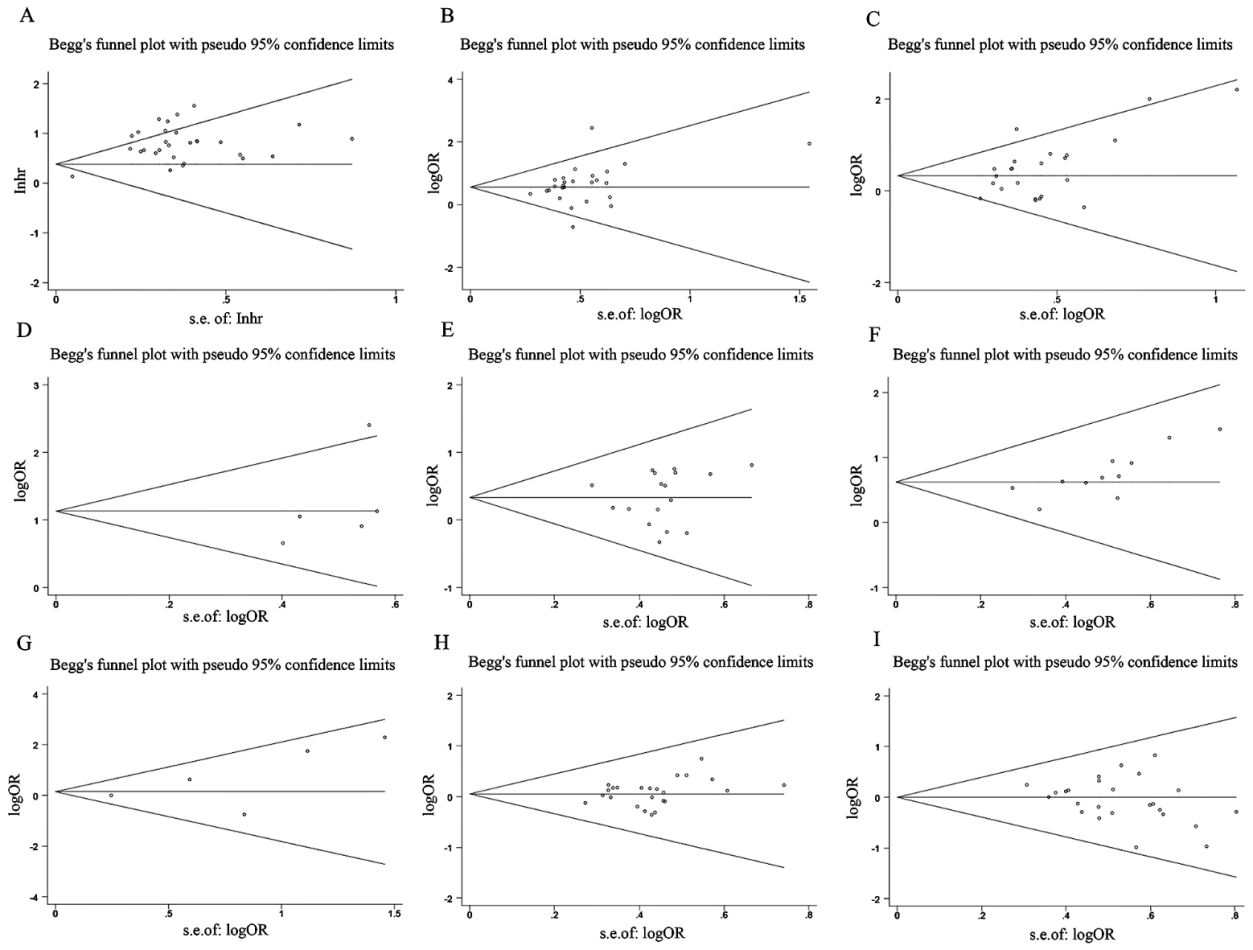
Funnel plot about the relationship between SNHG expression and survival outcome of Hepatocellular carcinoma (HCC). *Note* (**A**) OS; (**B**) TNM stage; (**C**) Tumor size; (**D**) LNM; (**E**) Histological grade; (**F**) Depth of invasion. (**G**) DM; (**H**) Age; (**I**) Gender

Outcomes of the sensitivity analysis indicated that, except for one paper (Lan T and Tu ZQ), the outcomes of the other papers did not have a considerable impact on the overall outcome. We used this information to conduct a subgroup analysis, and the findings demonstrated that the OS rate results had increased robustness and reliability after this paper was removed (Fig. 10). Begg’s test results indicated that except for invasion depth (Pr > |z| = 0.005), no OS publication bias was observed. (Pr > |z| = 0.206), TNM stage (Pr > |z| = 0.502), LNM (Pr > |z| = 0.851), DM (Pr > |z| = 0.086), tumor size (Pr > |z| = 0.051), histologic status (Pr > |z| = 0.650), age (Pr > |z| = 0.582), and gender (Pr > |z| = 0.269) (Fig. 11).

## Discussion

Despite not directly encoding proteins, lncRNAs regulate many tumor cell behaviors, including cell proliferation, apoptosis, drug resistance, immigration, and invasion affecting the progression of breast cancer [64], pancreatic cancer [65], and head and neck squamous cell carcinoma [66]. Many lncRNA SNHGs were found to be abnormally expressed in liver cancer [41, 63]. SNHG that is abnormally expressed has the potential to directly affect downstream signaling cascades or to function as a competitive endogenous RNA, absorbing microRNAs in a sponge-like fashion. The drug resistance, immigration, proliferation, and invasion of liver cancer cells are influenced by the indirect regulation of downstream signaling pathways or genes. In patients with cancer, there is a significant correlation between the DM, LNM, tumor size, TNM stage, PFS, and OS [27, 33, 42, 43, 48]. Multiple studies have demonstrated that members of the SNHG family have the potential to serve as therapeutic targets and prognostic indicators for liver cancer. Because SNHG expression was inconsistent with liver cancer prognosis in multiple prior studies, this study used a meta-analysis to comprehensively and systematically analyze the link between liver cancer patients’ prognosis and SNHG expression.

This investigation analyzed 38 relevant publications and found that liver cancer tissues expressed more SNHG family members. The combined HR and its 95%CI results showed that increased SNHG expression predicted poor liver cancer outcomes, including shorter OS and DFS duration. Inconsistent cut-off values, number of cases, follow-up time, and expression differences between different original literatures were taken into account. In this study, subgroup analysis findings showed that the mean, median, subgroup with > 100 cases, and subgroup with < 100 cases had follow-up times of at least 60 months. High SNHG expression substantially anticipated poor OS in these categories. Furthermore, elevated SNHG expression was predictive of easier LNM, advanced TNM stage, worse histologic grade, easier DM, and greater tumor size according to the pooled OR and its 95% CI values.

Many researchers tried to determine how SNHG affects liver cancer progression molecularly (Table 5). First, by directly affecting downstream signaling cascades or genes, SNHG may alter liver cancer cell biology. Zhang et al. [28] found that SNHG1 down-regulates p53 to increase HCC cell growth and block apoptosis. Zhang et al. [67] proposed that DANCR (SNHG13) could facilitate the proliferation, immigration, and invasion of Hep3B and HepG2 cells by interacting with PTEN signaling. By up-regulating p62 expression, Zhong et al. [58] demonstrated that SNHG16 can promote the growth, immigration, and infiltration of HuH-7 and HepG2 cells while preventing apoptosis. Secondly, by functioning as a sponging microRNA and an endogenous RNA competitor, SNHG can control downstream genes or signaling cascades. According to Meng et al. [27], SNHG1 can activate the FOXK1/Snail axis via sponging and down-regulating miR-376a, which in turn can drive the proliferation, invasion, immigration, and suppression of apoptosis in HCC cells. Li et al. [34] showed that SNHG5 may enhance GSK3β expression through sponging and down-regulating miR-26a-5p, which may aid in the processes of epithelial-mesenchymal transition, proliferation, invasion, and migration. Xie et al. proved that SNHG7 may contribute to the proliferation and block apoptosis of Hep3B and HepG2 through the down-regulation of Bax and caspase-3 by down-regulating miR-9-5p [36]. Xie et al. [51] observed that SNHG16 may promote matrix metalloproteinase (MMP) 2 and MMP9 expression through sponging and reduce miR-195, which could aid in the proliferation and invasion of HCC cells. Third, certain members of the SNHG family may have a considerable impact on liver cancer cells’ resistance to medications related to tumors. According to Zhang et al. [38], SNHG3 may enhance HCC cell invasion, proliferation, and sorafenib resistance by down-regulating miR-128 and up-regulating CD151 expression. Liu et al. reported that DANCR facilitates Sorafenib resistance of HCC cells by activating interleukin 6/STAT3 signaling. Jing et al. [43] showed that SNHG16 may contribute to the sorafenib resistance through the interaction with early growth response 1 by sponging and down-regulating miR-23b-3p. Finally, SNHG family members may affect HCC cell autophagy to enhance liver cancer progression. To stimulate HCC cell proliferation, and migration, and prevent apoptosis and autophagy, Huang et al. [42] found that SNHG11 up-regulated argonaute-2 via down-regulating miR-184. SNHG family members that have reduced expression in liver cancer tissues may have a better survival outcome for patients with HCC. For example, Hu et al. [30] found that lncGAS5 up-regulates miR-21 to suppress HCC cell invasion and migration. Yang et al. [63] revealed that lncGAS5 inhibits cell invasion of HCC cells via up-regulating reversion-inducing cysteine-rich protein with Kazal motifs (RECK) by targeting miR-135b.

**Table 5:** Regulation mechanism of SNHG involved in hepatocellular carcinoma cancer cells

Author and year	lncSNHG	expression level	role	miR-RNA	Downstream genes or pathways	cell lines	function (high SNHG expression)
Meng FZ [21]	SNHG1	upregulation	oncogene	miR-376a	FOXK1/Snail axis	HL7702,HepG2,SMMC-7721 and HuH-7	induce proliferation, invasion and migration, suppress apoptosis
Zhang M [28]	SNHG1	upregulation	oncogene	-	p53	SMMC-7721, MHCC97H, HCCLM3 and HepG2	promotes cells proliferation, inhibits apoptosis
Zhang PF 2018 [38]	SNHG3	upregulation	oncogene	miR-128	CD151	PLC/PRF/5, Hep3B, HepG2, MHCC97L, Huh7, SMMC-7721, and HCCLM3	promotes HCC cell invasion, induces EMT and sorafenib resistance
Li YR 2018 [34]	SNHG5	upregulation	oncogene	miR-26a-5p	GSK3β	Hep3B, HepG2, SMCC-7721, MHCC-97 L, MHCC-97 H, Huh7 and LO2	induce proliferation, invasion and migration and EMT process
Chen SY 2019 [68]	SNHG6	upregulation	oncogene	miR-let-7c-5p	c-Myc	MHCC-97 H and HCC-LM3	promotes proliferation
Cao C 2016 [32]	SNHG6	upregulation	oncogene	miR-26a/b	TAK1	BEL-7402, SMMC-7721, MHCC-97 H, SK-Hep-1, Huh7 and HCC-LM3	promoting cellular proliferation and inhibiting apoptosis
Fan XX [23]	SNHG6	upregulation	oncogene	miR-6509-5p	HIF1A	Hep3B and Huh7	promote proliferation, migration and invasion
XieYT 2020 [36]	SNHG7	upregulation	oncogene	miR-9-5p	CNNM1,bcl-2, bax, caspase-3	THLE-3, BEL-7404, HCCLM3, Hep3B and HepG2	facilitated cell proliferation, suppressed cell apoptosis
Yang X [37]	SNHG7	upregulation	oncogene	miR-122-5p	RPL4	Huh7, Hep3B, HCCLM3, MHCC97 H	induce cell proliferation, migration and invasion
Dong JY 2018 [69]	SNHG8	upregulation	oncogene	miR-149-5p	E-cadherin, N-cadherin, and Vimentin	LO2, Huh6, Huh7, SK-hep1, HepG2, and PLC5	facilitated cell proliferation, invasion, and Migration
Zhao ZB 2021 [16]	SNHG7	upregulation	oncogene	miR-122-5p	FOXK2,E-cadherin, N-cadherin and Vimentin	SNU449, Hep3B, and THLE-2	induce cell proliferation and migration
Feng SG [33]	SNHG9	upregulation	oncogene	miR-23a-5p	miR-23a-5p/Wnt3a Axis	HUH6,HepG2, QSG7701	facilitated cell proliferation
Lan T [45]	SNHG10	upregulation	oncogene	miR-150-5p	c-Myb	SNU-182, Huh-7, Hep3B, SK-Hep1, and SNU-387	promote cell proliferation, invasion, and migration and EMT process
Huang W [42]	SNH11	upregulation	oncogene	miR-184	AGO2	HL-7702, SK-HEP-1, Hep G2, HuH-7, and Li-7	induce Proliferation, Migration, inhibit Apoptosis and Autophagy
Lan T [44]	SNHG12	upregulation	oncogene	miR-199a/b-5p	MLK3,NF-κB pathway	SK-Hep1	induced cell proliferation and suppress cell apoptosis
Wang X [50]	DANCR	upregulation	oncogene	miR-222-3p	ATG7	Bel7407, Hep3B, HepG2, Huh7 and MHCC97H	accelerate cell proliferation and inhibit autophagy
Liu Y [48]	DANCR	upregulation	oncogene	-	IL-6/STAT3 Signaling	HEK-293T, Huh7, Huh7/sorafenib-resistant (SR) and Hep3B/ SR and Hep3B	facilitate Sorafenib Resistance
Yuan SX [54]	DANCR	upregulation	oncogene	miR-214, miR- 320a, miR-199a	CTNNB1	293T, SMMC7721	increased stemness features of HCC cells
Zhang H [56]	SNHG14	upregulation	oncogene	-	PTEN signaling	Hep3B and HepG2 cells	promoted cell proliferation, migration, and angiogenesis
Xu XY [52]	SNHG14	upregulation	oncogene	miR-217	E2F3	THLE-2, Huh-7, Hep3B	induce cell proliferation and suppress cell apoptosis
Liao ZB [46]	SNHG14	upregulation	oncogene	miR-876-5p	miR-876-5p/SSR2	HepG2 and Hep3B	promoted proliferation and metastasis
Lin RX 2021 [71]	SNHG14	upregulation	oncogene	miR-206	SOX9	MHCC97-H, Bel-7404, HepG2, SMCC7721, and QGY-7703	contribute to the proliferation, invasion, and migration
Chen W 2020 [40]	SNHG15	upregulation	oncogene	miR-18b-5p	LMO4	BEL-7402, HepG2, SMMC-7721, Hep3B	promote cell proliferation, invasion and migration and inhibit apoptosis
Dai W [41]	SNHG15	upregulation	oncogene	miR-490-3p	HDAC2	HuH-1, HuH-7 and L-O2	facilitate cell proliferation, migration and invasion
Jing Z [43]	SNHG16	upregulation	oncogene	miR-23b-3p	EGR1	ATCC HB-8064, ACTT	promote Sorafenib Resistance
Lin Q 2018 [47]	SNHG16	upregulation	oncogene	miR-4500	STAT3	SMMC-7721, L02, MHCC‐97 H, HepG2	promoting cell proliferation, migration, invasion, and EMT process as well as inhibiting cell apoptosis
Zhong JH 2019 [58]	SNHG16	upregulation	oncogene	-	p62	HuH-7, HepG2, SMMC-7721, HL-7702	promoted proliferation, migration, and invasion, while inhibiting apoptosis
Hu YL 2020 [70]	SNHG16	upregulation	oncogene	miR-605-3p	TRAF6/NF-κB feedback loop	HCCLM3, MHCC97L and MHCC-97 H	promoted proliferation, migration
Ye JF [53]	SNHG16	upregulation	oncogene	miR-140-5p	-	HepG2/SOR	facilitate Sorafenib Resistance
Xie XH [51]	SNHG16	upregulation	oncogene	miR-195	MMP-2, MMP-9	SMMC7721 and HepG2	promote proliferation, invasion
Zhu XM [59]	SNHG17	upregulation	oncogene	-	-	HepG2 and SNU-182	promotes cell proliferation and migration
Zhang DY 2016 [55]	SNHG20	upregulation	oncogene	-	-	HL-7702, MHCC-97 H, HepG2, SK-Hep-1, SMMC-7721, and BEL-7402	promoted proliferation, migration and invasion
Liu JX [60]	SNHG20	upregulation	oncogene	-	ZEB1, ZEB2, N-cadherin, E-cadherin and Vimentin	MHCC97L, SMCC- 7721, MHCC97H and Huh-7	induce cell proliferation and invasion
Zhang YX [62]	SNHG22	upregulation	oncogene	miR-16-5p	DNMT1	Huh7, HCCLM6, MHCC97H and SNU-398	promoted cell proliferation, invasion and migration
Luo J [61]	MEG8 (SNHG23)	upregulation	oncogene	miR-367-3p	TGFβR1	HepG2, Huh7, HCCLM3, and HMCC-97 H	promoted cell proliferation, invasion and migration

This study inevitably has certain limitations. Initially, the results of this research could only be relevant to Asian or Chinese populations, as all of the patients included in the study were from China. Second, the survival prognosis’s HR value and its 95% CI are explicitly provided in a few of the included studies, while others only provide the number of patients and survival curve. The software Engauge 4.0 version was applied to obtain the HR value indirectly, which is inevitable There may be some statistical bias or other bias. Third, the overall results may be subject to a certain level of bias due to inconsistent sample sizes, statistical analysis methods, follow-up duration, cut-off values, and other factors among different original studies. We conducted a subgroup analysis to mitigate these biases. Nevertheless, this research is the first meta-analysis to investigate the relationship between SNHG family member expression and HCC prognosis. Meanwhile, the molecular biological mechanism of SNHG affecting the progression of liver cancer was also comprehensively summarized.

## Conclusion

Most SNHG family members have substantial expression in HCC tissues, and high expression is positively connected with poor OS, advanced TNM stage, easy LNM and DM, poorer histopathological grade, and greater tumor size. SNHG may be an effective HCC prognostic marker and potential therapeutic target.

## Data Availability

This manuscript contains all study data or may be obtained from the corresponding author upon reasonable request.
